# T47. IS THERE A DIURNAL VARIATION IN PSYCHIATRIC SYMPTOMS IN SCHIZOPHRENIA?

**DOI:** 10.1093/schbul/sby016.323

**Published:** 2018-04-01

**Authors:** David Daniel, Alan Kott

**Affiliations:** Bracket Global, LLC

## Abstract

**Background:**

Anecdotally we have observed subgroups of schizophrenic patients who experienced a diurnal variation in a wide range of psychiatric symptomology. Such a pattern could have substantial ramifications in both clinical care and clinical trials. For example, two commonly used measures of symptom severity in clinical trials, the Positive and Negative Symptom Scale (PANSS) and the Negative Symptom Assessment Scale (NSA-16) contain numerous items that are rated based entirely or in part on observations of the subject during the interview. We hypothesized that inconsistency in the time of day of assessment in subjects whose symptoms were influenced by circadian rhythms could introduce an erratic “noise” element in the longitudinal measure of their symptoms.

To investigate this hypothesis we compared the change in PANSS total score and individual PANSS items across consecutive visits by whether the assessments had been conducted at consistent vs. inconsistent times of day.

**Methods:**

2109 individual subject visits from multiple schizophrenia clinical trials for which PANSS interview start time was available were included in the analysis. 1,764 pairs of consecutive PANSS interviews within individual subjects were identified and the time difference between the start times of the interviews were calculated. The absolute time difference was divided into quartiles and the first quartile (assessments least disparate in time) and the fourth quartile (assessments most disparate in time) were compared in the analyses. The mean absolute change in PANSS total and PANSS individual items between the consecutive visits was compared between the 2 groups using a t-test. Given the exploratory and hypothesis driven nature of the analysis we did not correct for multiple comparisons.

**Results:**

The average absolute difference in interview start times was 79.5 minutes. The group with assessments closest in time (N = 446) had their interviews start no later than 30 minutes apart while the group with assessments most disparate in time (N = 445) had their interview start with at least 170 minutes difference. Of the 30 PANSS items, items P4 (Excitement 0.33 vs. 0.44, p =0.0077), P7 (Hostility 0.31 vs. 0.47, p < 0.001), N5 (Difficulty in abstract thinking 0.28 vs. 0.35, p = 0.043), N7 (Stereotyped thinking0.26 vs. 0.35, p = 0.021) G1 (Somatic concern 0.34. vs. 0.43, p = 0.041), G2 (Anxiety 0.42 vs. 0.53, p = 0.019), G6 (Depression 0.38 vs.0.52, p = 0.0044), G7 (Motor retardation 0.28 vs. 0.37, p = 0.025), and G8 (Uncooperativeness 0.29 vs. 0.39, p = 0.02) had an absolute difference significantly higher in the disparate group than in the close group. The mean absolute difference in the PANSS total was 5.1(+/-5.8) in the close group and 5.9(+/-5.4) in the disparate group, (p = 0.08).

**Discussion:**

The results demonstrate a statistically significant effect of variation in time of day on symptom severity. These findings underscore the risk of potential noise (erratic changes) in longitudinal assessment of symptom severity when ratings are done at different times of day. Moreover, it suggests that symptom severity assessed in a standard PANSS interview may not generalize over the entire day, much less over the standard one week rating period. This is important because many PANSS items are rated partially or entirely on the interview which may not be a representative “biopsy” of the subject’s mental state or behavior during the one week rating period. This highlights the potential remedies of more frequent or ecological measurements.

Hufford et al (2014) demonstrated a statistically significant effect of variation in the time of day on signal from the MCCB cognitive battery. Our results confirm our anecdotal observations that severity of non-cognitive schizophrenic symptoms are impacted by circadian rhythms as well.

